# Supplementary material for: Inhibiting ALK2/ALK3 Signaling to Differentiate and Chemo-Sensitize Medulloblastoma
Source: Cancers (Basel). 2022 Apr 22;14(9):2095. doi: 10.3390/cancers14092095 (PMC9102092; doi:10.3390/cancers14092095)
Supplement: Supplementary file 1 [file cancers-14-02095-s001.zip › cancers-1653912-supplementary.pdf]

cancers

MDPI

Inhibiting ALK2/ALK3 Signaling to Differentiate and Chemo-Sensitize Medulloblastoma

Doria Filipponi, Marina Pagnuzzi-Boncompagni and Gilles Pagès

Supplemental Materials and Methods

1.1. Migration assays

Both DAOY and HD-MB03 cells were cultured overnight without serum overnight. 70 000 DAOY or 250 000 HD-MB03 cells were plated in serum-free medium with 30nM LDN-193189 (Selleckchem, Houston, TX, USA) into the upper compartment of wells (24-well 8.0 µm pore size, Corning, Dutscher). The lower chamber was supplemented with 10% serum-medium. After 30 min, 20ng/ml of BMP4 (SRP3016, Sigma Aldrich, St. Louis, MO, USA) was added into the upper compartment for 24h at 37 °C in 5% CO2. Cells were fixed with 3% paraformaldehyde and stained with 0.4% Crystal Violet. Bright field images were taken with a 4× objective Evos XL Core Cell Imaging system (Thermo Fisher, Waltham, Massachusetts, United States). Cells migration was quantified using ImageJ (NIH) software.

1.2. Patients RNA expression data, DNA methylation analysis and overall survival

For patients expression data (figure 2B-C, supplemental figure 6B) and DNA methylation analysis, we used public datasets deposited on "R2: Genomic Analysis and Visualization Platform expression data: https://hgserver1.amc.nl/cgi-bin/r2/main.cgi (Accessed on 2022/2/12) and provided tools were used to generate all graphics and statistical analysis. Kaplan-Meier curves, for overall survival analysis of patients were generated using R2 Cavalli database (https://hgserver1.amc.nl/cgi-bin/r2/main.cgi) (Accessed on 2022/2/12). For Kegg pathway analysis we used the portal: https://david.ncifcrf.gov/ (Accessed on 2022/2/12).

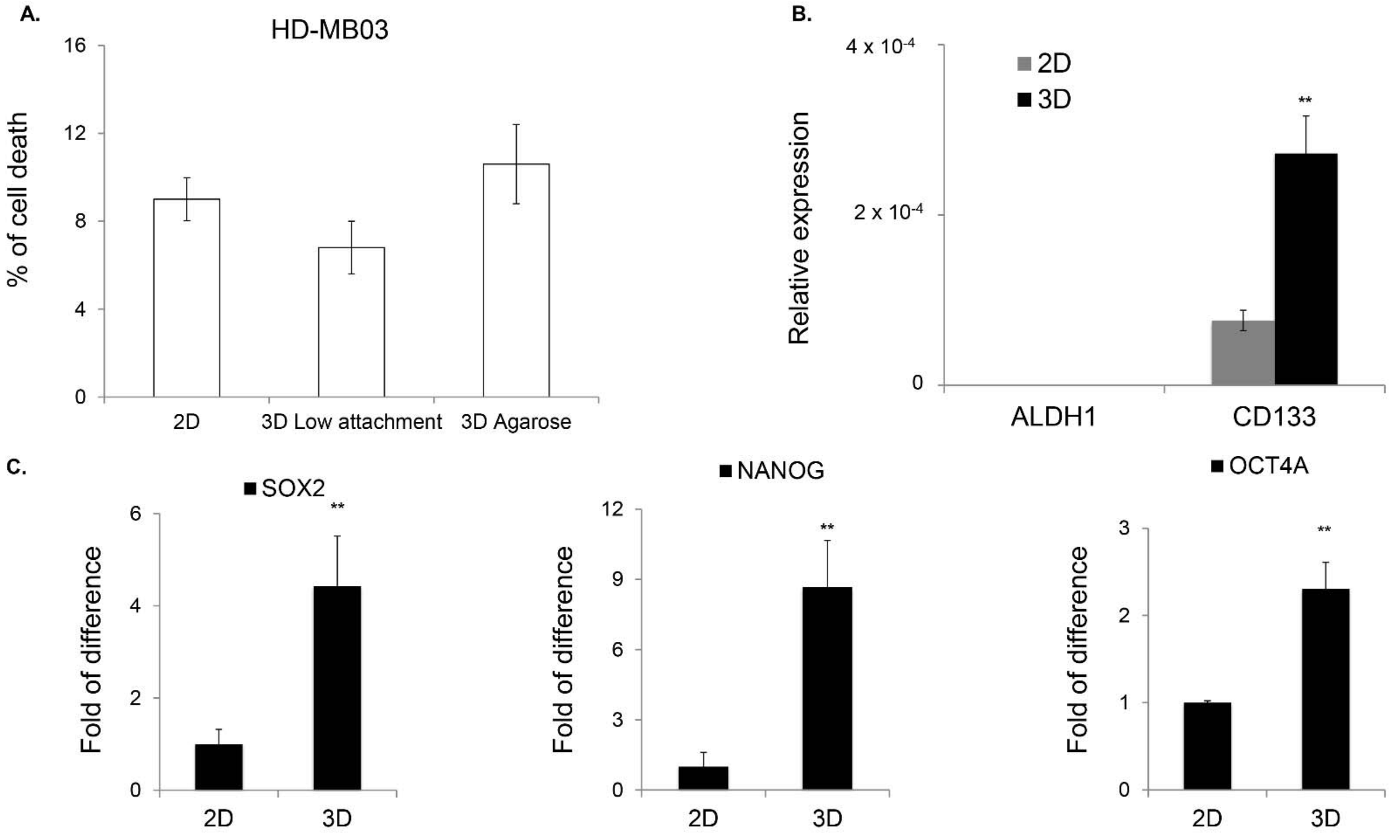

Figure S1. Tumorsphere(s) generated in low attachment plates. (A) Cell viability of HD-MB03 cells measured as trypan blue exclusion in adherent cells (2D) and spheroid generates in classical low attachment plates or agarose-noble coated plates. B) qRT-PCR analysis of brain CSCs-related markers, shows the relative expression of CD133 and ALDH1 in 2D and 3D cells generated from HD-MB03 using the standard low attachment protocol. (n= 3 biological replicates). $*p < 0.05$; $**p < 0.01$; $***p < 0.001$ Two-tailed paired Student's t test. Data are mean ± SD. C) qPCR analysis of pluripotent transcription factors SOX2, NANOG and OCT4A, in HD-MB03 spheres (3D) generated using standard low attachment plates relative to adherent counterpart (2D). $*p < 0.05$; $**p < 0.01$; $***p < 0.001$. Two-tailed paired Student's t test. Data are mean ± SD.

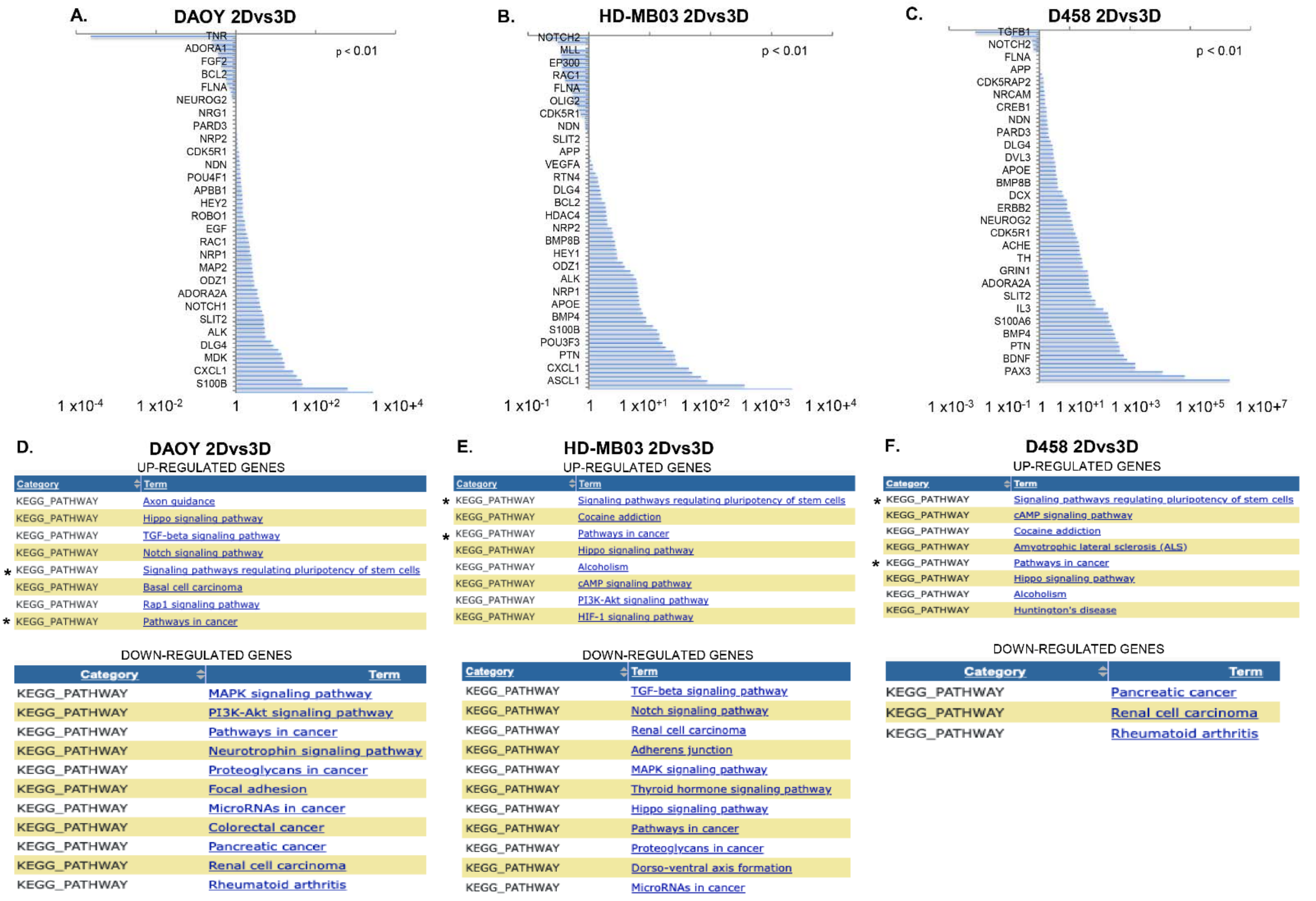

Figure S2. RT2 profiler PCR arrays in tumorsphere(s) generated from MB cell lines.

(A-C) RT2 profiler PCR arrays for DAOY, HD-MB03 and D458, shows the differentially regulated genes in 3D related to 2D, expressed as fold of difference and normalized to the expression of housekeeping genes ACTB. $p<0.01$ (D-F) KEEG pathways analysis of signaling induced in 3D for DAOY, HD-MB03 and D458 as indicated.

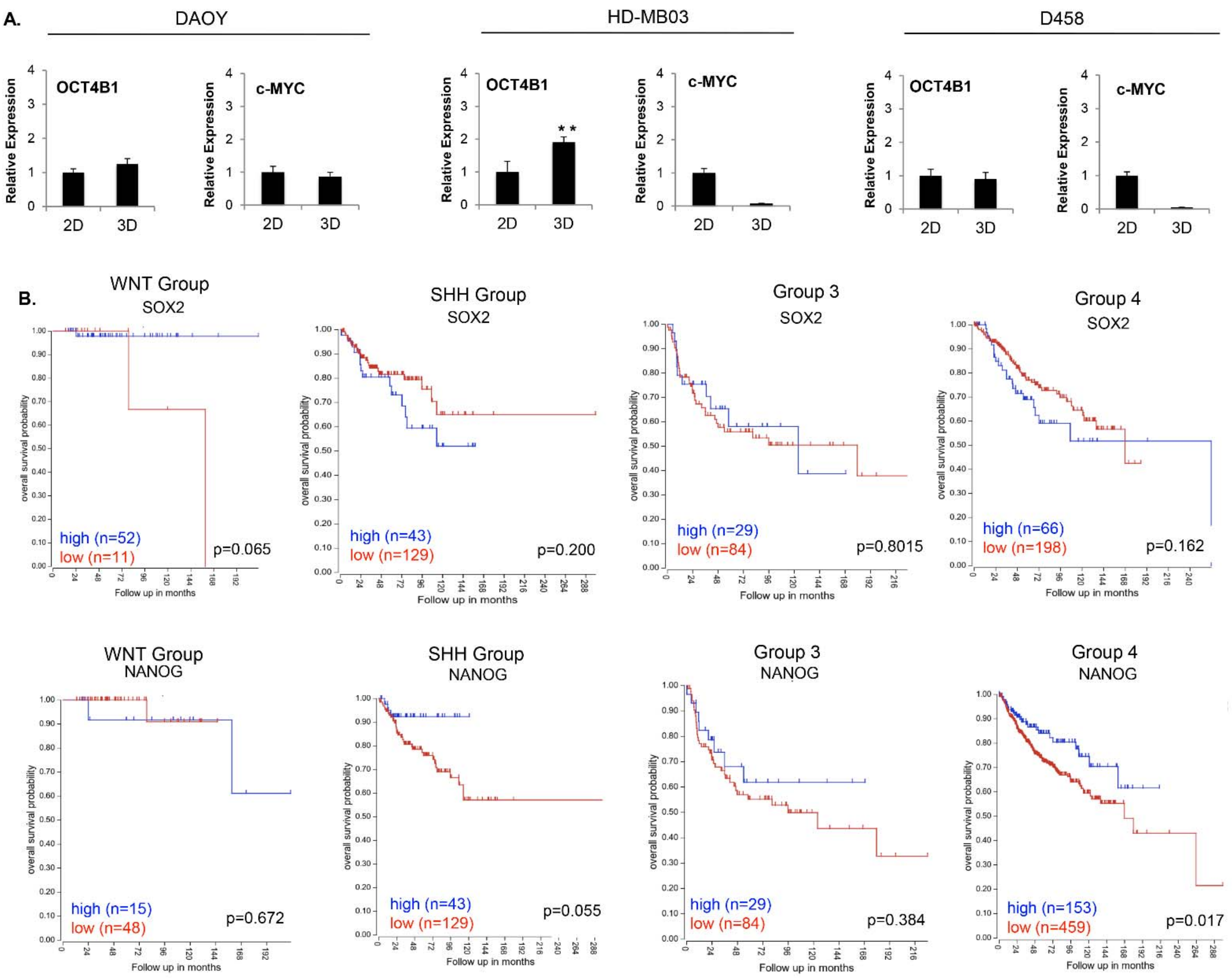

Figure S3. Overall survival of patients for pluripotent transcription factors.

(A) qPCR analysis of transcription factors OCT4B1 and c-MYC in spheres (3D) generated from DAOY, HD-MB03 and D458 lines relative to adherent counterpart (2D). *$p < 0.05$; **$p < 0.01$; ***$p < 0.001$. Two-tailed paired Student's t test. Data are mean ± SD. (B) Kaplan-Meier curves show the analysis of overall survival of patients generated from R2 Cavalli database (https://hgserver1.amc.nl/cgi-bin/r2/main.cgi) for high (blue) and low (red) SOX2 expressing patients (A) and for high (blue) and low (red) NANOG expressing patients (B) for each MB sub-group. The relative p value is shown.

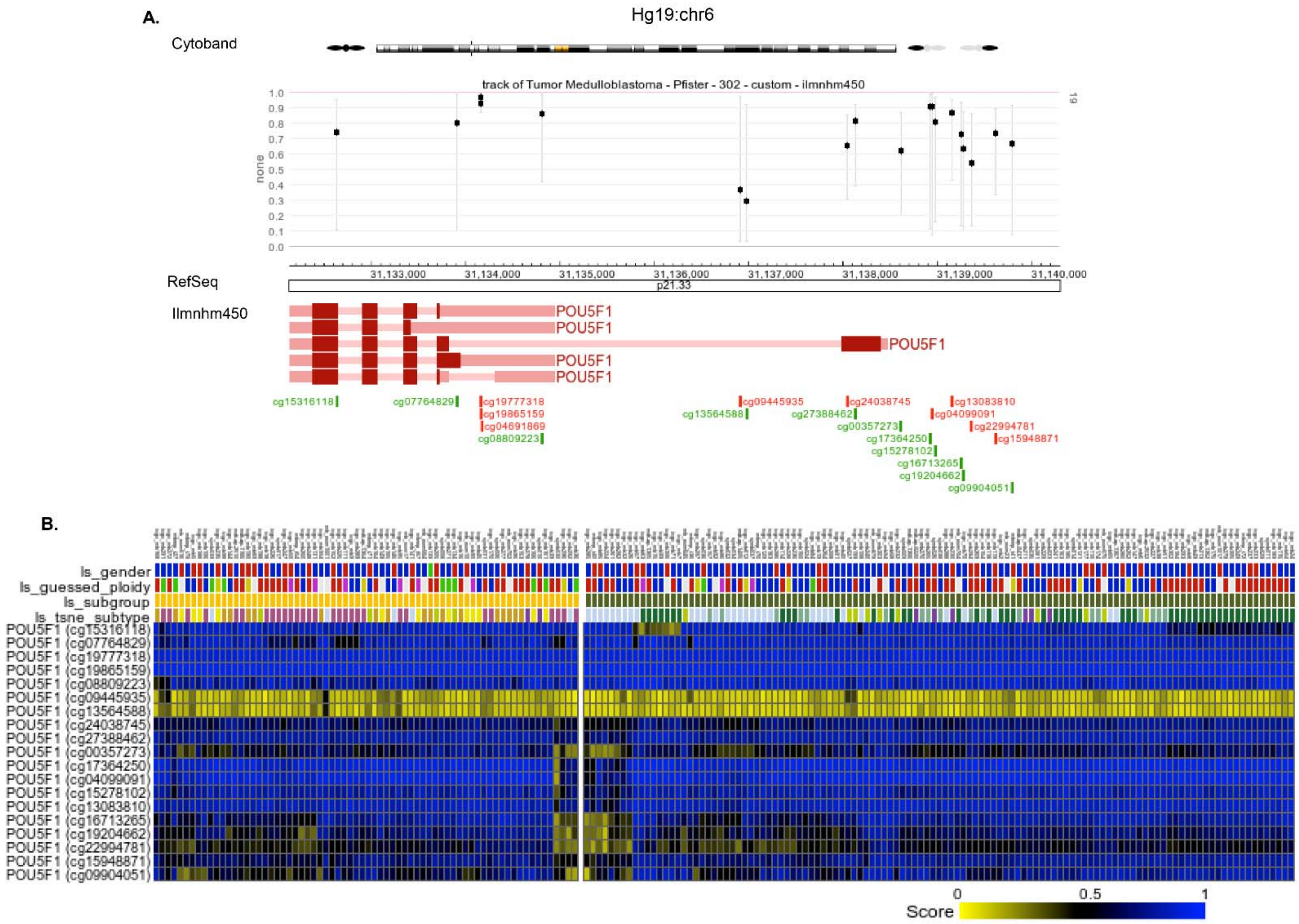

Figure S4. DNA methylation profile of OCT4 locus in MB patients.

(A) Genome browser representation showed the alignment of the probes used for DNA methylation analysis along the human OCT4 locus. (B) Heat map of DNA methylation analysis across methylation database generated from human patients (Pfster-302 database). The methylation level is expressed as b-value ranging from 0 (yellow) to 1 (blue).

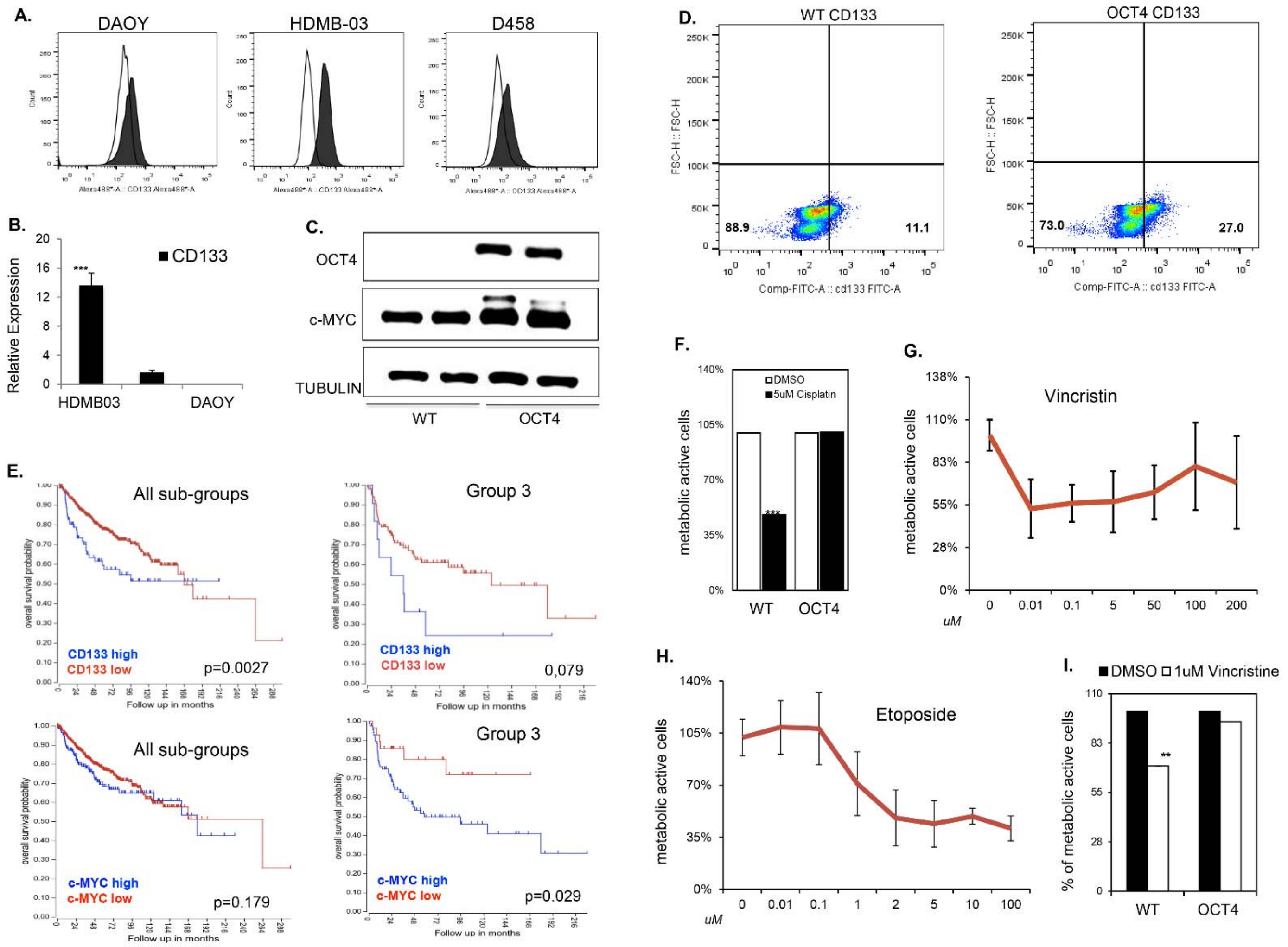

Figure S5. OCT4 overexpression promotes TICs, tumorigenicity potential and drug resistance.

(**A**) FACS analysis shows the expression of CD133 in DAOY, HD-MB03 and D458 cells. (**B**) qPCR analysis shows the expression of CD133 in parental HD-MB03, DAOY, D458. *p < 0.05; **p < 0.01; ***p < 0.001. Two-tailed paired Student's t test. Data are mean ± SD. (**C**) Immuno blot analysis shows the expression level of OCT4 protein and c-MYC in parental and OCT4 overexpressing HD-MB03 cells. Tubulin has been used as a loading control. (**D**) FACS analysis shows the level of CD133 in parental (WT) and OCT4 over-expressing (OCT4) HD-MB03 cells with or without antibody. (**E**) Kaplan-Meier curves show the analysis of overall survival of patients generated from R2 Cavalli database (https://hgserver1.amc.nl/cgi-bin/r2/main.cgi) for high (blue) and low (red) CD133 OR c-MYC expressing patients in all medulloblastoma sub-group and group 3 respectively. The relative p-value is showed. (**F**) MTT assay shows the response to 5μM cisplatin treatment of wild-type (WT) and OCT4-expressing HD-MB03 (OCT4) relative to the respective DMSO control. (**G**) Dose response curve of vincristine and etoposide treatment in MTT assay for HDMB03. (**H**) Dose response curve of Etoposide 10nM-100μM treatment in MTT assays for HD-MB03. (**I**) Cytotoxic effect analyzed in MTT assays of vincristine in parental (WT) and OCT4 over-expressing (OCT4) HD-MB03 related to DMSO treated counterpart (DMSO).

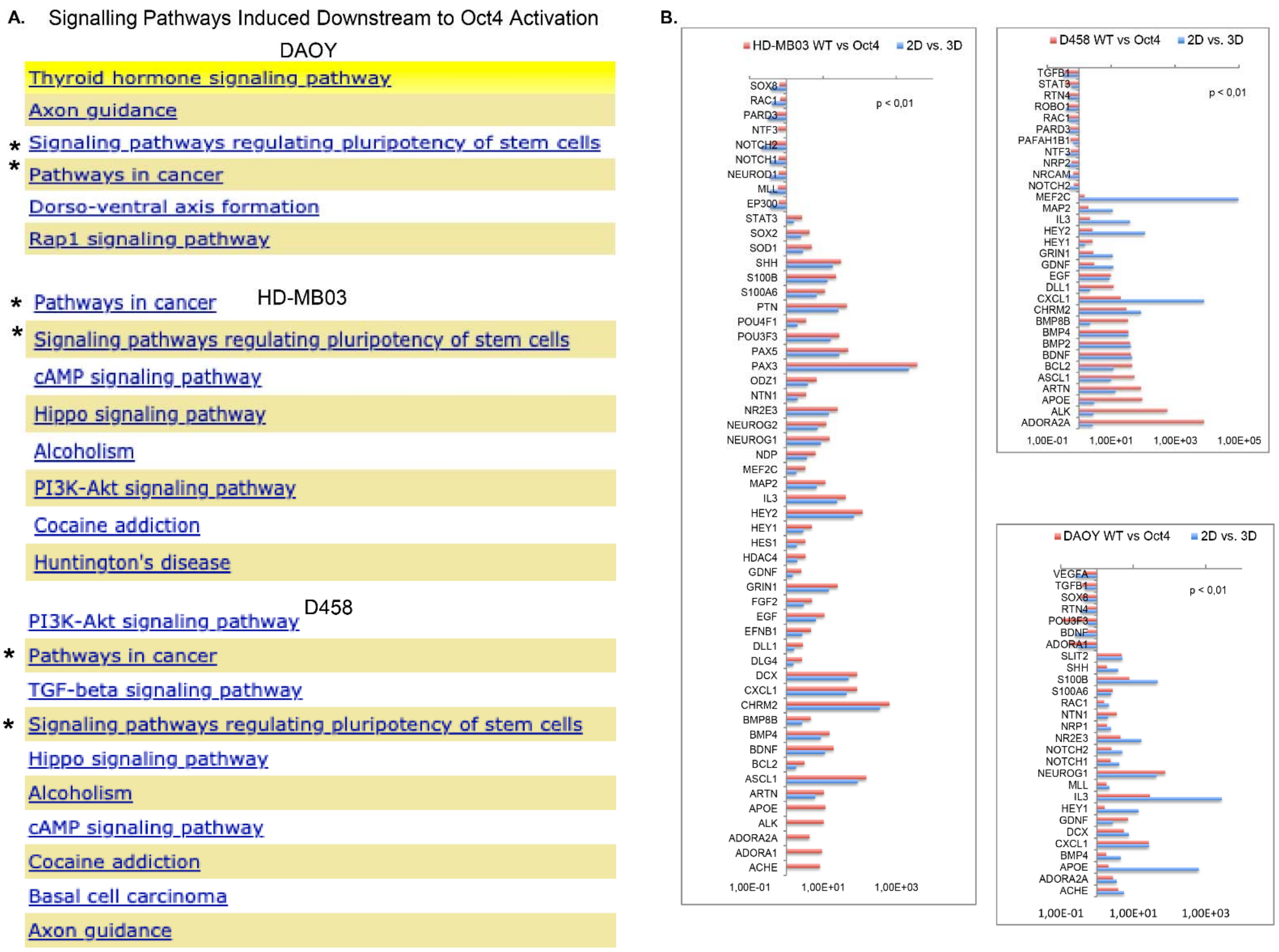

Figure S6. RT2 profiler PCR profile shows the overlapping genes between tumorsphere(s) and OCT4 over-expressing MB cells.

(A) KEEG pathways analysis shows the signaling pathways induced downstream to OCT4 activation in all tree cell lines as indicated. (B) Overlapping of the differentially regulated genes in OCT4 over-expressing (OCT4) related to parental cells (2D) and in spheres (3D) related to parental (2D) cells for each cell lines as showed. RT2 profiler PCR arrays showed the common differentially regulated genes between the three different cell lines as indicated. The gene expression level is showed as a fold of difference in 3D relative to 2D and normalized to the expression of housekeeping genes ACTB. **$p<0.01$.

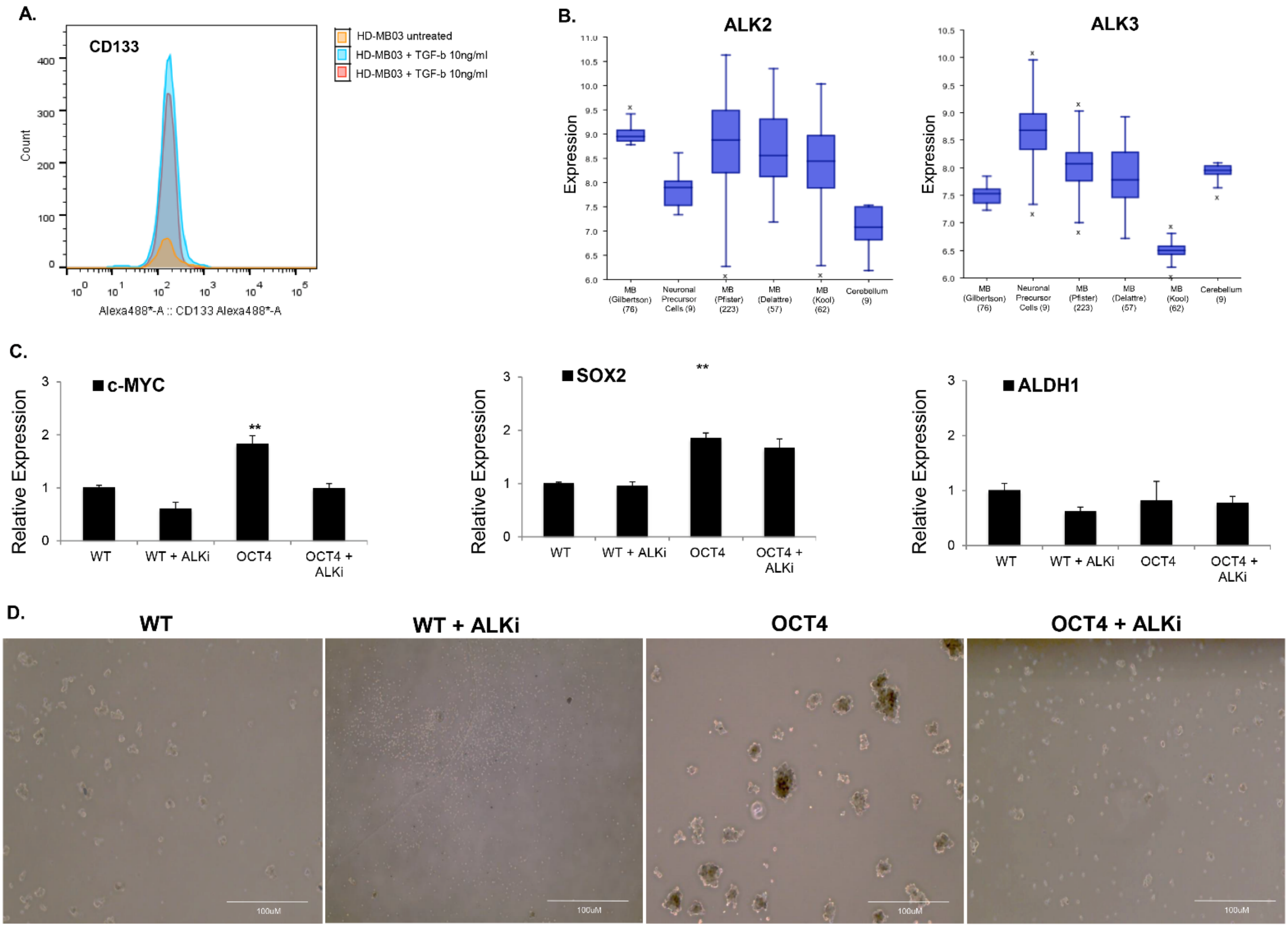

Figure S7. The ALK2/3 inhibitor, LDN 198836, decreases TICs and tumorigenic potential.

(A) FACS analysis show the expression of CD133 in HD-MB03 untreated (orange) or treated with 10ng/ml or TGF-b for 24h (light blue) or 48h (pink). (B) Box dot plots show the expression level of ALK2, ALK3 in normal cerebellum (n=9) from Roth database, MB patients: M1 (n=76) from Gilbertson database, M2 (n=51) from den Boer database, M3 (n=223) from Pfister database, M4 (n=57) from Delattre database, M5 (n=62) from Kool database and embryonic stem cells, ES (n=6) from Viale database. (C) qPCR analysis shows the expression of c-MYC, SOX2, ALDH1 in parental HD-MB03 (WT) and OCT4-expressing (OCT4) HD-MB03 cells treated or untreated with ALK2/3 inhibitor (LDN 198836) for 24h. *$p < 0.05$; **$p < 0.01$; ***$p < 0.001$. Two-tailed paired Student's t test. Data are mean ± SD (D) Representative images of tumor sphere generated from parental (WT) and OCT4 over-expressing (OCT4) HD-MB03 cells treated or untreated with ALK2/3 inhibitor (LDN 198836).

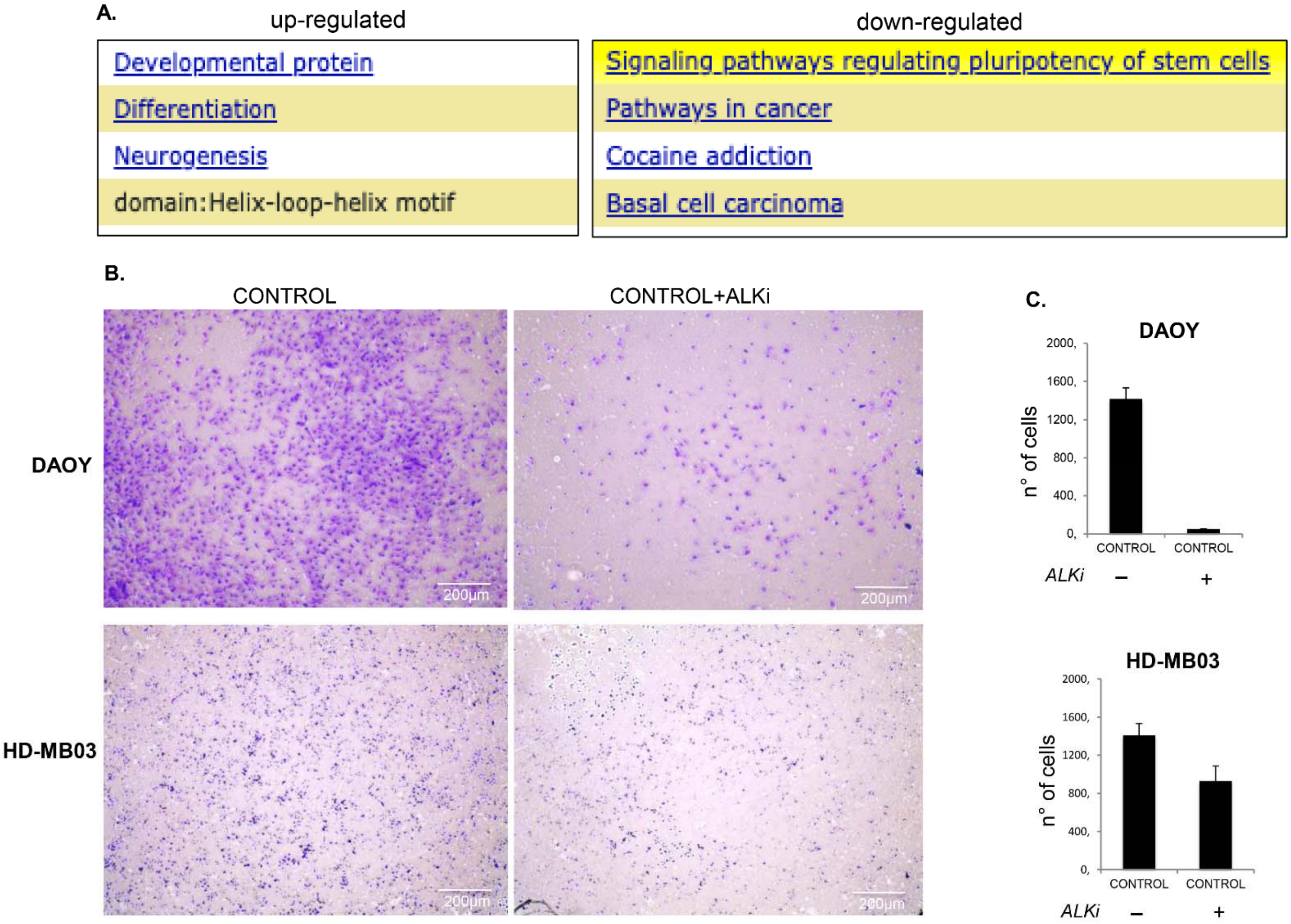

Figure S8. The ALK2/3 inhibitor, LDN 183366, decreases the migration ability of MB cells. (A) KEEG pathway analysis shows the up-regulated (left panel) and down-regulated signaling pathway (right-panel) in HD-MB03 cell lines treated with ALK2/3 inhibitor (LDN 198836). (B) Boyden chambers assay in DAOY and HD-MB03 cell lines shows the effects of LDN 183366 on migration. Quantification of migrations assay expressed as percentage of number of migrating cells.

Related to Figure 2D

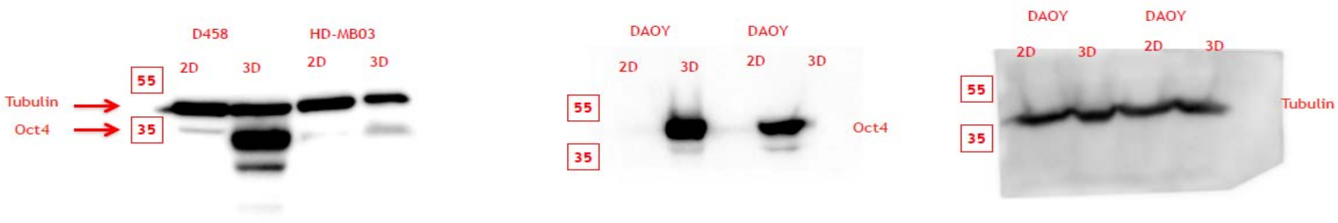

Related to Figure 3A

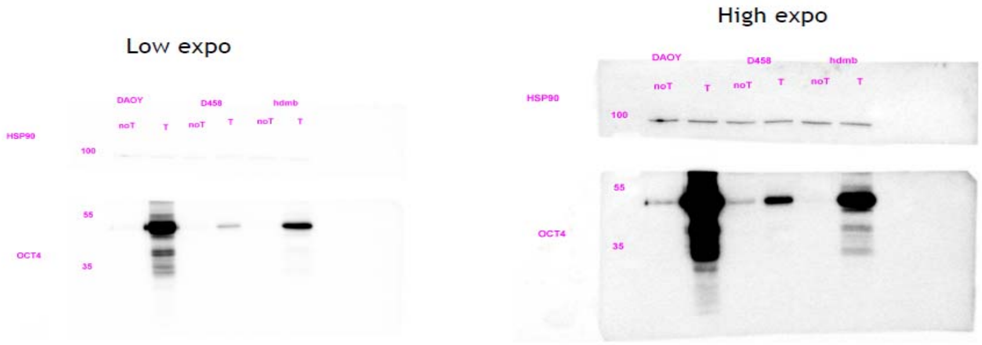

Related to Figure 5A

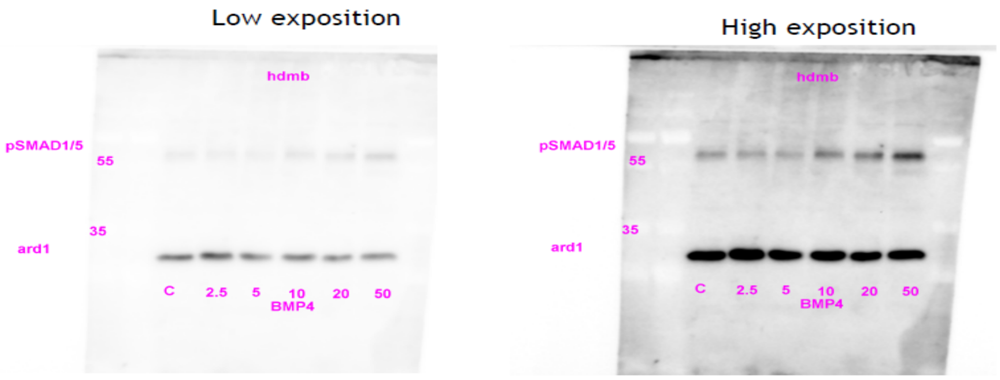

Related to Figure 5D

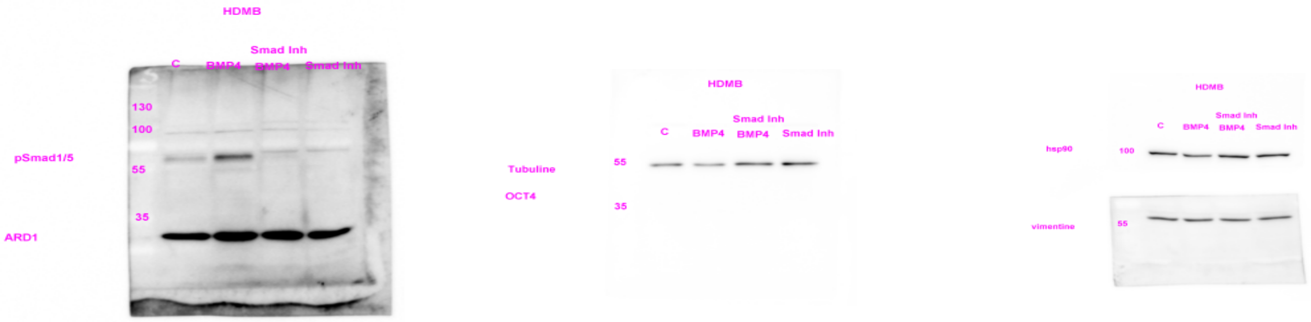

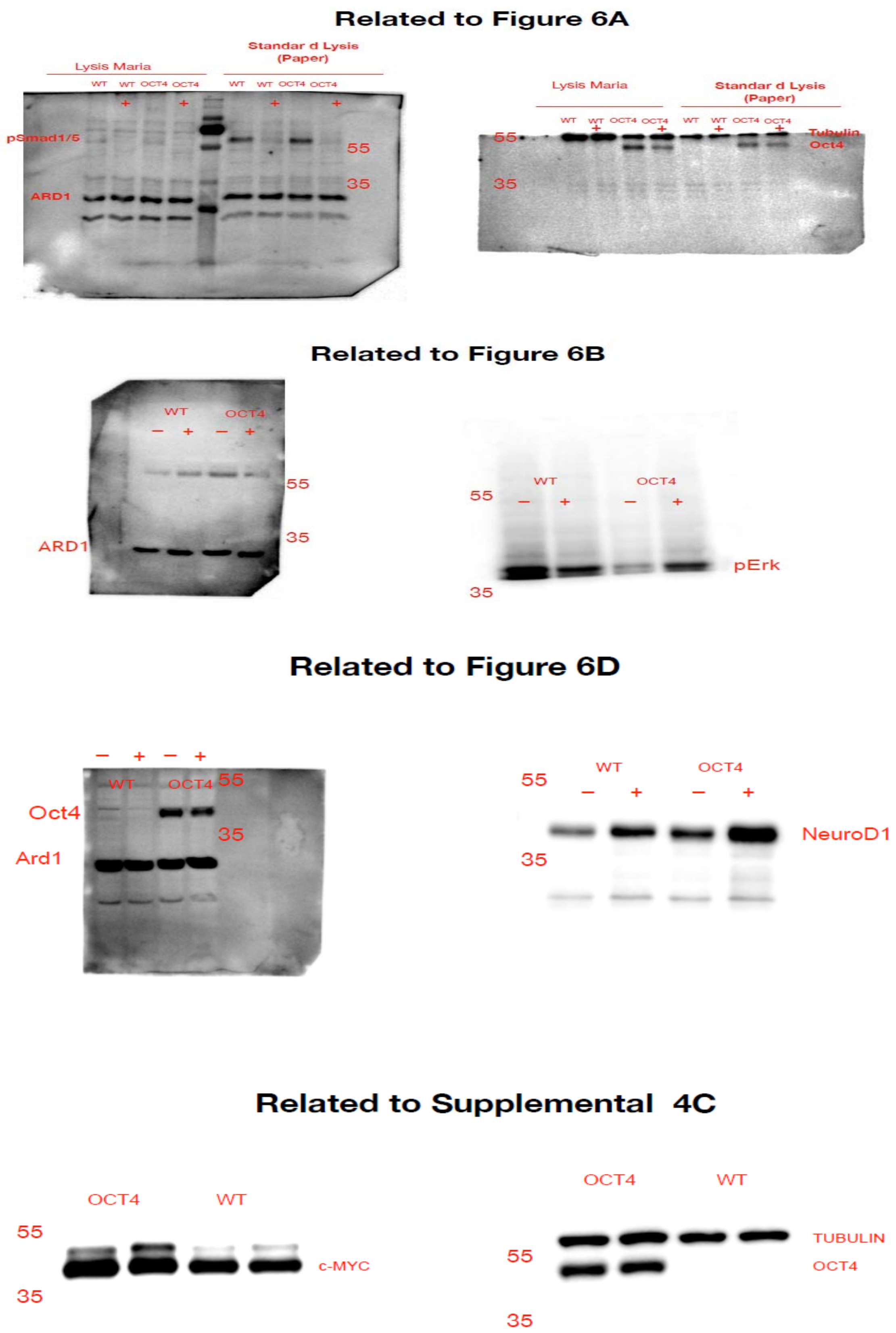

Figure S9. The original blots.

Table S1. RT_PCR Primers used in this study.

OCT4A FOR	CGCAAGCCCTCATTTCAC
OCT4A REV	CATCACCTCCACCACCTG
GAPDH FOR	CCATGACCCCTTCATTGACC
GAPDH REV	GACAAGCTTCCCGTTCTCAG
TUBULIN FOR	GCGAGATGTACGAAGACGAC
TUBULIN REV	TTTAGACACTGCTGGCTTCG
NANOG FOR	ATTCAGGACAGCCCTGATTCTTC
NANOG REV	TTTTTGCGACACTCTTCTCTGC
SOX2 FOR	GAGCTTTGCAGGAAGTTTGC
SOX2 REV	GCAAGAAGCCTCTCCTTGAA
KLF4 FOR	TCCCACATGAAGCGACTTCC
KLF4 REV	GTCTCTCTCCGAGGTAGGGG
CD133 FOR	AAGCATTGGCATCTTCTATGG
CD133 REV	AAGCACAGAGGGTCATTGAGA
ALDH1A FOR	TGTTAGCTGATGCCGACTTG
ALDH1A REV	TTCTTAGCCCGCTCAACACT
OCT4B1 FOR	TCCCTCTCCCTACTCCTCTTC
OCT4B1 REV	TTCTATTTGGTGCGTTCC
c-MYC FOR	AATGAAAAGGCCCCCAAGGTAGTTATCC
c-MYC REV	GTCGTTTCCGCAACAAGTCCTCTT
